# A Strong Case for Prudent School Siting: The West Fertilizer Company Explosion

**DOI:** 10.1289/ehp.124-A187

**Published:** 2016-10-01

**Authors:** Carrie Arnold

**Affiliations:** Carrie Arnold is a freelance science writer living in Virginia. Her work has appeared in *Scientific American*, *Discover*, *New Scientist*, *Smithsonian*, and more.

At 7:51 P.M. on 17 April 2013, an explosion rocked the town of West, Texas, when fertilizer-grade ammonium nitrate stored at the West Fertilizer Company blew up with the explosive force of 12.5 tons of TNT.^1^ Fifteen people died, and 260 were injured. More than 150 offsite buildings were destroyed by the blast and subsequent fire, among them a middle school, a high school, a nursing home, and an apartment complex. An investigation by the Chemical Safety Board (CSB) concluded that if the explosion had occurred during the day, casualties likely would have been much higher and would have included a significant number of children.^1^

**Figure:**
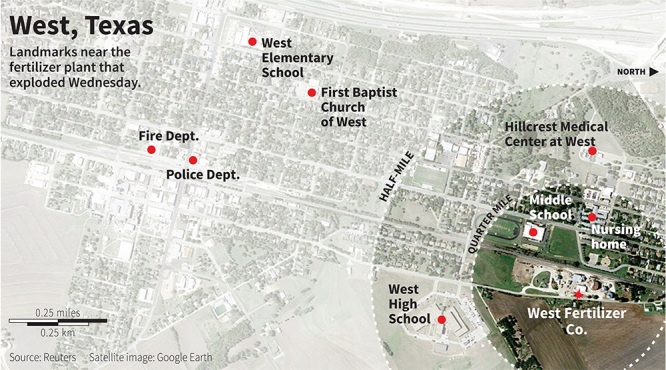
The U.S. Environmental Protection Agency has issued a nonbinding recommendation that no schools be built within a half-mile of hazardous facilities. The two schools destroyed in West, Texas, fell within that radius. © Reuters Graphics

Veronica Tinney, a recommendations specialist at CSB, and her colleagues reviewed the data from the investigation and discovered that the close proximity of schools to potentially hazardous facilities was not unique to West. In this issue of *EHP*, they present a set of broad recommendations for schools and community members to manage this risk.^2^ “The dangers from this explosion aren’t an isolated risk,” Tinney says. “It’s a widespread problem, and we got lucky in West that school was not in session.”

What immediately struck CSB investigators when they arrived on the scene was the close proximity of so many community buildings to the storage facility, Tinney says. When the West Fertilizer Company opened for business in 1962, its buildings were located some distance from the town. Over the years, however, as the local population grew, settlements began encroaching upon the area around the facility.

When it comes to building new schools, the U.S. Environmental Protection Agency has recommended that no schools be built within a half-mile of hazardous facilities, but compliance with this guidance is voluntary.^3^ Like many states, Texas has no regulations about siting schools near chemical storage sites or other hazardous areas.

The CSB’s formal investigation ultimately traced the explosion to an uncontrolled fire in a building that housed fertilizer-grade ammonium nitrate. The board further found that, in Texas alone, nearly half of the 40 facilities that store fertilizer-grade ammonium nitrate are within a half-mile of a school, some even closer than the schools destroyed in the West explosion.

It is difficult, if not impossible, to determine precisely how many children have been harmed at school from chemical incidents at nearby facilities, says Ayana Anderson, a public health analyst at the Agency for Toxic Substances and Disease Registry (ATSDR). That is because not all states collect such data, and for those that do, there is no one single surveillance system.

However, Anderson adds, ATSDR data can provide some insight into quantifying and characterizing chemical releases in schools. She explains that the agency captures data on injury types, evacuations, incidences of sheltering in place, sites where chemicals are released, and types of chemicals released. Using data from the Hazardous Substances Emergency Events Surveillance system, ATSDR epidemiologists found that 11% of reported injuries in chemical incidents between 1999 and 2008 were in students at school.^4^

Tinney and colleagues encourage the creation of local and state rules about zoning and school siting to better protect students. For existing schools situated near hazardous chemical facilities, the authors recommend that school district leaders work closely with emergency responders to understand what dangers exist and how to respond in the event of an emergency.

In a 2016 analysis of ammonium nitrate explosions,^5^ safety expert Vyto Babrauskas of Fire Science and Technology, Inc., wrote that relatively simple measures, such as improving preventive measures employed at storage facilities, can make a big difference in safety. “Companies need to store ammonium nitrate in fireproof buildings with sprinklers,” he says. “As long as it isn’t ignited in an uncontrolled fire, there’s no explosion danger.”
